# The Peroxisome Proliferator-Activated Receptor *γ* Agonist Pioglitazone Protects Vascular Endothelial Function in Hypercholesterolemic Rats by Inhibiting Myeloperoxidase

**DOI:** 10.1155/2020/1845969

**Published:** 2020-01-07

**Authors:** Dapeng Zhang, Yehong Wang, Ming Yi, Suli Zhang, Ye Wu

**Affiliations:** ^1^Beijing Key Laboratory of Metabolic Disorders Related Cardiovascular Diseases, Capital Medical University, Beijing 100069, China; ^2^Heart Center & Beijing Key Laboratory of Hypertension Disease, Beijing Chaoyang Hospital, Capital Medical University, Beijing 100020, China; ^3^Department of Physiology, Shanxi Medical University, Taiyuan 030001, Shanxi, China; ^4^Department of Gastroenterology, Shuozhou People's Hospital, Shuozhou 036002, Shanxi, China; ^5^Department of Physiology and Pathophysiology, School of Basic Medical Sciences, Capital Medical University, Beijing 100069, China

## Abstract

**Objective:**

Hypercholesterolemia- (HC-) induced endothelial dysfunction is the first step of atherogenesis, and the peroxisome proliferator-activated receptor *γ* (PPAR*γ* (PPAR*γ* (PPAR*γ*) has been reported to attenuate atherosclerosis formation; however, the underlying mechanisms are not fully understood. The present study was designed to determine whether myeloperoxidase (MPO) mediates HC-induced endothelial dysfunction and the role of the PPAR*γ* agonist pioglitazone (PIO) in attenuating endothelial dysfunction.

**Methods:**

Male Wistar rats were fed with normal or high cholesterol diets for 8 weeks. HC rats were randomized to receive dapsone (DDS, the MPO inhibitor) during the last 6 days or PIO for the remaining 4 weeks. Vascular endothelial function was determined by comparing vasorelaxation to ACh, an endothelium-dependent vasodilator, and SNP, an endothelium-independent vasodilator in vascular rings in vitro. The vascular MPO activity, NO_*x*_ content, and cGMP level were measured by the MPO activity assay kit, NO assay kit, and cGMP RIA kit.

**Results:**

Compared with rats fed with normal diet, endothelium-dependent vasodilation, NO_*x*_ content, and cGMP level were decreased, and MPO activity was increased in thoracic aortas of rats fed with HC diet. There was a negative correlation between vascular endothelial function, NO_*x*_ content or cGMP level, and MPO activity. PIO obviously reduced the MPO activity, increased NO_*x*_ content and cGMP level, and improved endothelium-dependent vasodilation function in HC rats, which was essentially the same as that seen with DDS. And, there was a negative correlation between vascular endothelial function, NO_*x*_ content or cGMP level, and MPO activity in the HC group and the PIO intervention group.

**Conclusion:**

MPO might provoke vascular endothelial dysfunction in hypercholesterolemic rats by reducing the NO biological activity and impairing the NO/cGMP/cGK signaling pathway. PIO might inhibit vascular MPO activity and increase NO bioavailability with the net result of reversing endothelial dysfunction.

## 1. Introduction

Coronary artery disease (CAD) becomes one of the most important diseases that affect longevity and survival quality of aging [1]. Endothelial dysfunction is the first stage in the progression of atherogenesis [2], and hypercholesterolemia is one of the most important causes of endothelial dysfunction [3]. The mechanism of vascular endothelial dysfunction caused by hypercholesterolemia is complex, in which a decrease in the bioavailability of nitric oxide (NO) [4] and impaired NO/cGMP/cGK signaling are considered important contributory mechanisms [5]. Therefore, if the cause responsible for decreased NO bioavailability in hypercholesterolemia is determined and then blocked, it is thought that vascular endothelial function could be effectively maintained, thereby reducing the occurrence of atherosclerosis.

Myeloperoxidase (MPO) is an oxidase that is stored in azurophilic granules of neutrophils and monocytes, which is released extracellularly during inflammation [6]. MPO plays an important role in the formation and development of many diseases, including atherosclerosis [7]. Studies have shown [8] that MPO is abundantly accumulated in the basement membrane under the vascular endothelium in hypercholesterolemia, and it is speculated that it may lead to endothelial dysfunction by the precipitation of NO. However, the specific mechanism of action of MPO remains to be elucidated.

Upon activation of peroxisome proliferator-activated receptor *γ* (PPAR*γ*), it plays a crucial role in the regulation of inflammation and improvement of vascular endothelial function [9], thereby regulating MPO gene expression [10]. However, whether PPAR*γ* agonists can restore NO bioavailability by regulating MPO, thereby improving vascular endothelial function and delaying the progression of atherogenesis in hypercholesterolemia, have not been confirmed.

Therefore, the aims of this investigation were as follows: first, to verify that vascular endothelial dysfunction is caused by a decrease in NO bioavailability in hypercholesterolemia, and on this basis, to observe and analyze whether MPO directs endothelial dysfunction in hypercholesterolemia by affecting the vascular NO/cGMP/cGK signaling pathway. We also aimed to further observe whether PPAR*γ* agonists could reverse vascular endothelial dysfunction in hypercholesterolemia and, if possible, to determine whether or not this was related to the regulation of vascular MPO and subsequent restoration of NO bioavailability.

## 2. Materials and Methods

### 2.1. Animals

All animal procedures utilized in the investigations conformed to the Guiding Principles in the Use and Care of Animals, published by the National Institutes of Health (NIH Publication No. 85-23, Revised 1996) and were approved by the Institutional Animal Care and Use Committee of Capital Medical University.

Healthy male Wistar rats weighing 110.0 ± 10.0 g (SPF grade) were purchased from Beijing Vital River Laboratory Animal Technology Co., Ltd, China. Animals were maintained in 12 h light-dark cycles, and food and water were available ad libitum. Before conducting the experiment, blood was drawn from the tail of each rat, and baseline plasma lipids were determined using assay kits (Nanjing Jiancheng Bioengineering Institute, China). Then, rats were randomly divided into two different dietary groups: the normal group (*n* = 12) was given a normal diet and the high-cholesterol (HC) group (*n* = 60) was given an HC diet, which comprised 1% cholesterol, 10% egg yolk powder, and 5% lard, for 8 weeks. Four weeks into the HC diet regimen, the rats' blood was redrawn and plasma lipid levels were determined. Thereafter, the rats in the HC group were randomly assigned to five groups: (1) HC group (*n* = 16); (2) HC + dapsone (DDS; the myeloperoxidase inhibitor, Sigma) group (*n* = 16): 100 *μ*mol/kg/day (dissolved in 1 ml/kg DMSO) for the last 6 days, intraperitoneal injection; (3) HC + DMSO (dimethyl sulfoxide) group (*n* = 6): 1 ml/kg/day for the last 6 days, intraperitoneal injection; (4) HC + pioglitazone (PIO, Zhejiang Huayi Pharmaceutical Co., Ltd., China) (*n* = 16): 10 mg/kg/day for the remaining 4 weeks, oral gavage; (5) HC + PIO + DDS group (*n* = 6): PIO (10 mg/kg/day) via oral gavage in the remaining 4 weeks, and DDS (100 *μ*mol/kg/day) via intraperitoneal injection in the last 6 days. At the end of the 8 weeks of the HC diet, the plasma lipid levels were determined again. All serum samples were collected from the 8 h fasted research subjects. Animals were euthanized by a physical method (decapitation, a suggested method for rodents by AVMA Guidelines on Euthanasia). All animals were euthanized with sodium pentobarbital (50 mg/kg, i.p.) to reduce animal anxiety on the guillotine and ensure euthanasia was rapidly accomplished to lessen the animal suffering. The thoracic aortic segments of all rats were collected for the detection of vascular endothelial function, MPO activity, NO content, and cGMP level.

### 2.2. Measurement of Vascular Endothelial Function

The thoracic aortic segments were excised and placed in ice-cold oxygenated HEPES buffer (mM: NaCl, 144; KCl, 5.8; MgCl_2_·6H_2_O, 1.2; CaCl_2_, 2.5; glucose, 11.1; HEPES, 5; pH 7.38–7.40), and adhering tissues were cleaned off and cut into rings (2 mm length) for the detection of vascular endothelial function. The endothelial function was determined as described previously [11]. Briefly, after the equilibration period, the artery segments were exposed to HEPES buffer containing 60 mM potassium (mM: NaCl, 144; KCl, 60; MgCl_2_·6H_2_O, 1.2; CaCl_2_, 2.5; glucose, 11.1; HEPES, 5; pH 7.38–7.40) until reproducible contractile responses were obtained. After washing with HEPES buffer, segments of thoracic aortas were precontracted with phenylephrine (PE, 10^−6^ mol/L, Sigma). Once a stable contraction was achieved, increasing concentrations of vasodilators were added to the chamber to obtain cumulative concentration-response curves. Endothelium-dependent dilation was measured by acetylcholine (ACh, 10^−9^–10^−5^ mol/L, Sigma), and endothelium-independent dilation was measured by sodium nitroprusside (SNP, 10^−10^–10^−6^ mol/L, Sigma).

### 2.3. Determination of Vascular MPO Activity

MPO activity in thoracic aortic tissue was measured using the MPO assay kit (Nanjing Jiancheng Bioengineering Institute, China) and calculated as U/g protein.

### 2.4. Detection of Total NO Content in Thoracic Aortic Tissue

NO has a short half-life and is oxidized to form NO_2_ and NO_3_*in vivo*. Thus, the detection of NO_*x*_ (+ NO_2_ + NO_3_) concentration has been demonstrated to reflect total NO formation. The NO_*x*_ content in thoracic aortic tissue was determined using the NO assay kit (nitrate reductase method) (Nanjing Jiancheng Bioengineering Institute, China) and calculated as nmol/mg protein.

### 2.5. Determination of cGMP in Thoracic Aortic Tissue

The cGMP levels in the thoracic aortic tissue were determined by [^125^I] cGMP radioimmunoassay with commercially available kits (Shanghai Chinese Medicine University, China) and assayed for cGMP in duplicates according to the manufacturer's instructions. The results of duplicate assays were averaged. The cGMP level was calculated as pmol/mg protein.

### 2.6. Statistical Analysis

Data were analyzed using SPSS19.0 software. Results are presented as mean ± SD. Comparisons between groups were made using one-way analysis of variance (ANOVA) followed by the Bonferroni post hoc test. The relationship was analyzed using linear regression. Differences were considered statistically significant at a value of *P* < 0.05.

## 3. Results

### 3.1. Vascular Endothelial Dysfunction in Hypercholesterolemic Rats

After eight weeks of a high cholesterol diet, levels of serum CHO, TG, and LDL-CHO were significantly higher than those in rats being fed a normal diet (Table 1), suggesting that the hypercholesterolemic rat model was successfully built.

**Figure 1:**
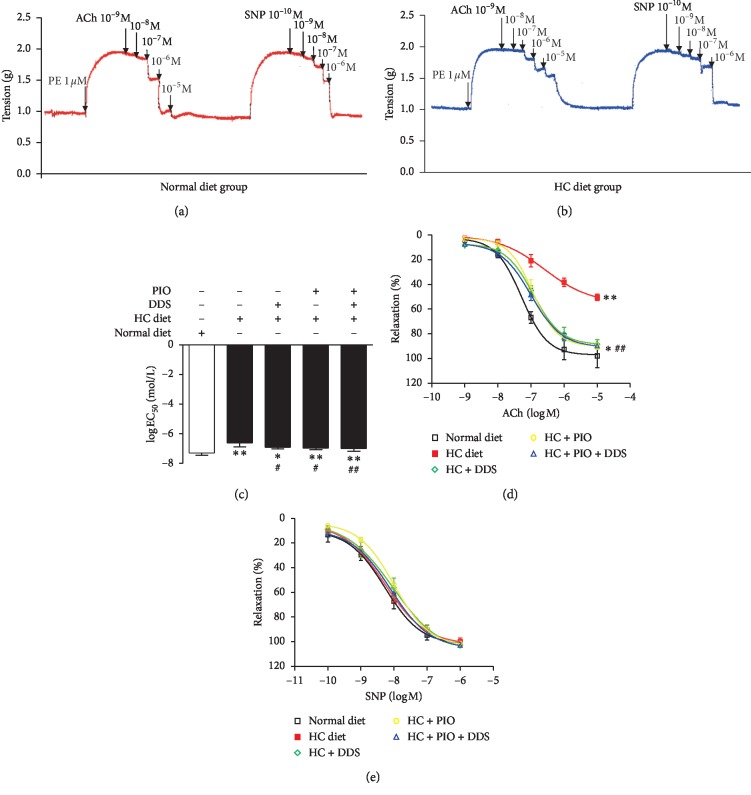
The vasodilatory response in thoracic aortic rings from different groups. The concentration-dependent curve induced by acetylcholine (ACh) and sodium nitroprusside (SNP) in thoracic aorta rings of normal diet rats (a) and hypercholesterolemic (HC) diet rats (b); the log EC_50_ value of vascular tone (c) and the concentration-dependent curve (d) induced by ACh in thoracic aorta rings with different groups; the concentration-dependent curve induced by SNP (e) in thoracic aorta rings with different groups. ^*∗*^*P* < 0.05 and ^*∗∗*^*P* < 0.01 vs. normal diet group; ^#^*P* < 0.05 and ^##^*P* < 0.01 vs. HC diet group. *n* = 6–10 rats/group.

After administration of acetylcholine (ACh) across a dose-dependent concentration gradient of 10^−9^–10^−5^ mol/L, the concentration-dependent vasodilatory response was seen in the thoracic aorta rings of normal diet rats (Figure 1(a)). The concentration-dependent curve induced by ACh in the thoracic aorta rings of hypercholesterolemic rats was severely shifted to the right, and the log EC_50_ of the vascular tone increased from −7.29 ± 0.16 mol/L to −6.61 ± 0.27 mol/L as compared with the normal control group (*P* < 0.01; Figure 1(c)). The maximum vasodilatation decreased from 97.88 ± 9.53% to 50.51 ± 2.44% (*P* < 0.01; Figures 1(b) and 1(d)). After administration of the exogenous NO donor sodium nitroprusside (SNP) at a cumulative concentration of 10^−10^ to 10^−6^ mol/L, there was no significant difference in the vascular tone when comparing both groups (Figures 1(a), 1(b), and 1(e)). SNPs are used to detect endothelium-independent relaxation responses of blood vessels. So, the results suggested evidence of vascular endothelial dysfunction in hypercholesterolemic rats.

### 3.2. The Vascular NO/cGMP/cGK Signaling Pathway Impaired and MPO Activity Decreased in Hypercholesterolemic Rats

NO is an important signaling molecule that is related to vascular endothelial function and exerts a vasodilatory effect through the NO/cGMP/cGK signaling pathway. To verify whether vascular endothelial dysfunction in hypercholesterolemic rats was associated with impairment of this signaling pathway, this study determined NO_*x*_ content and cGMP levels in vascular tissues, which reflected the biological activity of vascular NO at the cGMP level.

**Figure 2:**
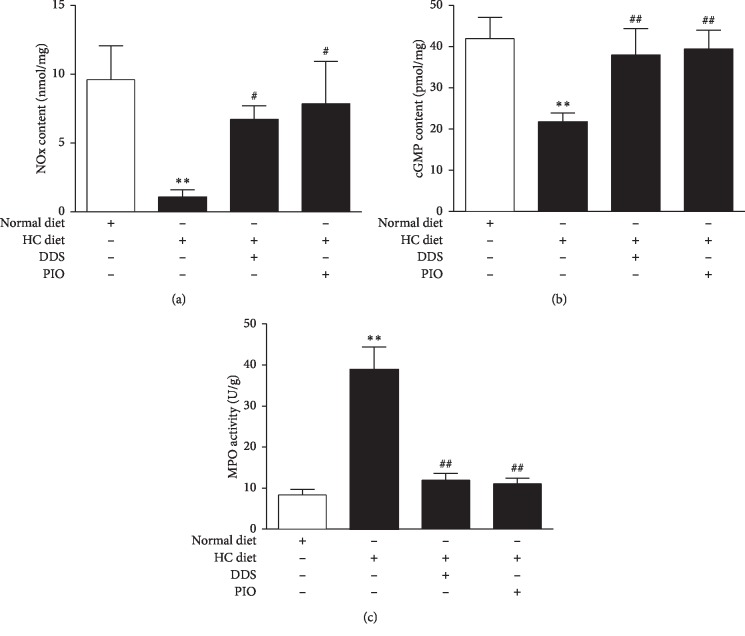
The changes of NO_*x*_ content, cGMP level, and MPO activity in thoracic aorta tissue with different groups: (a) NO_*x*_ content; (b) cGMP level; (c) MPO activity. ^*∗∗*^*P* < 0.01 vs. normal diet group; ^#^*P* < 0.05 and ^##^*P* < 0.01 vs. HC diet group. *n* = 6–10 rats/group.

As compared with normal diet rats, the content of vascular NO_*x*_ in hypercholesterolemic rats had decreased from 9.61 ± 2.47 nmol/mg to 1.09 ± 0.49 nmol/mg (*P* < 0.01; Figure 2(a)), and the cGMP level had decreased from 41.94 ± 5.18 pmol/mg to 21.81 ± 2.11 pmol/mg (*P* < 0.01; Figure 2(b)). This observation suggested that the biological activity of NO in hypercholesterolemic rats had decreased, and the vascular NO/cGMP/cGK signaling pathway was impaired. At the same time, this study found that the vascular MPO activity in hypercholesterolemic rats was approximately 4.5-folds higher than that of normal diet rats (8.37 ± 1.31 U/mg vs. 38.83 ± 5.56 U/mg, *P* < 0.01; Figure 2(c)).

### 3.3. The MPO Activity of Vascular Tissues Negatively Correlated with Vascular Endothelial Function, NO_*x*_ Content, and cGMP Level in Hypercholesterolemic Rats

**Figure 3:**
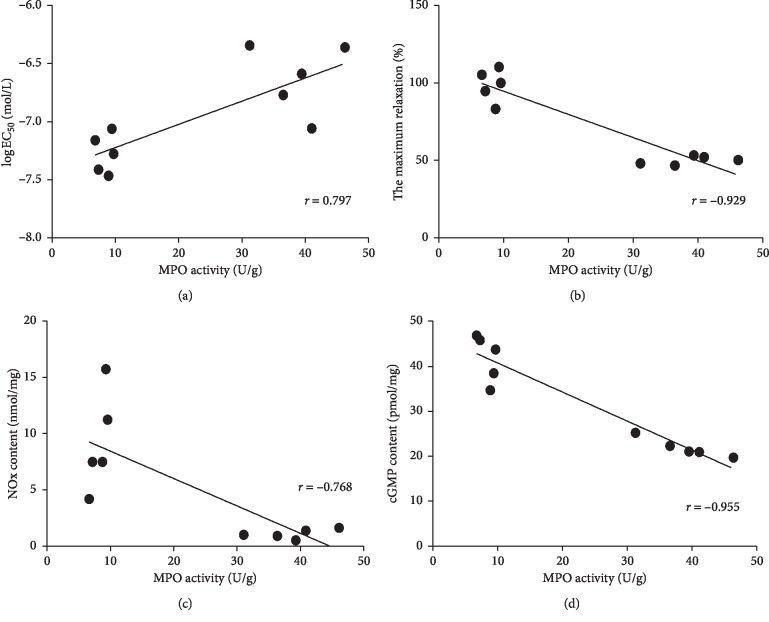
Correlation analysis of MPO activity with vascular endothelial function, NO_*x*_ content, and cGMP level in thoracic aorta rings between normal diet and HC diet groups. There was a positive correlation between MPO activity and the log EC_50_ value of ACh-induced vasodilation (a); ACh-induced maximum vasodilatation (b), NO_*x*_ content (c), and cGMP level (d) showed negative correlation with MPO activity.

We analyzed the association of vascular endothelial function and key signaling molecules (NO_*x*_ and cGMP) of the NO/cGMP/cGK pathway with MPO activity to explore whether MPO played a role in vascular endothelial dysfunction in hypercholesterolemia. The results showed that the log EC_50_ value of ACh-induced vasodilation positively correlated with MPO activity (*r* = 0.797, *P* < 0.01; Figure 3(a)); i.e., the EC_50_ value negatively correlated with the MPO activity. Similarly, ACh-induced maximum vasodilatation showed a negative correlation with MPO activity (*r* = -0.929, *P* < 0.01; Figure 3(b)). Moreover, vascular NO_*x*_ content and the cGMP levels negatively correlated with MPO activity (*r* = −0.768, *P* < 0.01; *r* = −0.955, *P* < 0.01; Figures 3(c) and 3(d)).

### 3.4. After Inhibiting the MPO Activity, Vascular Endothelial Dysfunction in Hypercholesterolemic Rats Alleviated, and the Impaired Vascular NO/cGMP/cGK Pathway Improved

To further investigate the role of MPO in hypercholesterolemia-induced endothelial dysfunction, the MPO inhibitor dapsone (DDS) was administered to hypercholesterolemic rats. After DDS intervention, the vascular MPO activity in hypercholesterolemic rats decreased from 38.83 ± 5.56 U/mg to 11.92 ± 1.63 U/mg (*P* < 0.01; Figure 2(c)), which did not affect blood lipid levels of treated rats (Table 1). There was no change in the solvent DMSO group (Supplementary Figure 1(a)).

As compared with the hypercholesterolemia group, we found that after DDS was administered to hypercholesterolemic rats, the vasodilation curve had significantly shifted to the left after administration of ACh at a cumulative concentration of 10^−9^ mol/L–10^−5^ mol/L. The vascular tone log EC_50_ value had decreased from −6.61 ± 0.27 mol/L to −6.91 ± 0.11 mol/L (*P* < 0.05; Figure 1(c)), and the maximum vasodilatation increased from 50.51 ± 2.44% to 88.42 ± 3.54% (*P* < 0.01; Figure 1(d)). After administration of SNP at a cumulative concentration of 10^−10^ mol/L–10^−6^ mol/L, there was no significant difference in vascular tone when comparing both groups (Figure 1(e)). In addition, there was no change in the solvent DMSO group (Supplementary [Supplementary-material supplementary-material-1]).

As compared with the hypercholesterolemia group, the vascular NO_*x*_ content increased from 1.09 ± 0.49 nmol/mg to 6.71 ± 0.98 nmol/mg (*P* < 0.05) after DDS intervention in hypercholesterolemic rats (Figure 2(a)). The cGMP level increased from 21.81 ± 2.11 pmol/mg to 37.97 ± 6.39 pmol/mg (*P* < 0.01; Figure 2(b)), while the solvent DMSO group remained unaltered (Supplementary Figures [Supplementary-material supplementary-material-1] and [Supplementary-material supplementary-material-1]). These observations suggested that MPO might be closely related to vascular endothelial dysfunction in hypercholesterolemic rats and played a key role in impairing the NO/cGMP/cGK signaling pathway by inhibiting NO biological activity.

### 3.5. After Intervention with the PPAR*γ* Agonist, Vascular Endothelial Dysfunction in Hypercholesterolemic Rats Alleviated, Vascular MPO Activity Decreased, and the NO/cGMP/cGK Signaling Pathway Improved

TZDs (thiazolidinediones, insulin sensitizers) are PPAR*γ*-selective agonists with high affinity, and pioglitazone (PIO) is a key example of one of these agonists. After four weeks of intragastric administration of PIO in hypercholesterolemic rats, blood lipid levels were significantly decreased (Table 1).

As compared with the hypercholesterolemia group, the concentration-dependent vasodilation curve that was induced by ACh had significantly shifted to the left after PIO intervention, and the vascular tone log EC_50_ value decreased from −6.61 ± 0.11 mol/L to −6.95 ± 0.12 mol/L (*P* < 0.05; Figure 1(c)); meanwhile, maximum vasodilatation increased from 50.51 ± 2.44% to 89.99 ± 2.68% (*P* < 0.01; Figure 1(d)), and there was no significant difference observed when comparing changes that were seen after DDS intervention (Figures 1(c) and 1(d)). Moreover, this study found that even after interventions with PIO and DDS, there was no significant difference in ACh-induced vasodilation in hypercholesterolemic rats as compared with that seen after intervention with PIO alone (Figures 1(c) and 1(d)). Thus, there was no more superimposed medication. There was also no significant difference in SNP-induced vasodilation when comparing between groups (Figure 1(e)).

In addition, when comparing the hypercholesterolemia group, the vascular MPO activity decreased from 38.83 ± 5.56 U/g to 11.05 ± 1.43 U/g (*P* < 0.01; Figure 2(c)) after PIO intervention—an observation that was similar to changes seen after DDC intervention. At the same time, when comparing with the hypercholesterolemic rats, it was found that the vascular NO/cGMP/cGK signaling pathway had also improved after PIO intervention, and the NO_*x*_ content increased from 1.09 ± 0.49 nmol/mg to 7.86 ± 3.07 nmol/mg (*P* < 0.05; Figure 2(a)); the cGMP level increased from 21.81 ± 2.11 pmol/mg to 39.47 ± 4.52 pmol/mg (*P* < 0.01; Figure 2(b)), which was essentially the same as that seen with DDS. These observations suggested that PIO could improve vascular endothelial dysfunction in hypercholesterolemic rats and did so by reducing MPO activity and increasing NO bioavailability.

### 3.6. The MPO Activity of Vascular Tissues Negatively Correlated with Vascular Endothelial Function, NO_*x*_ Content, and cGMP Level after Intervention with the PPAR*γ* Agonist

**Figure 4:**
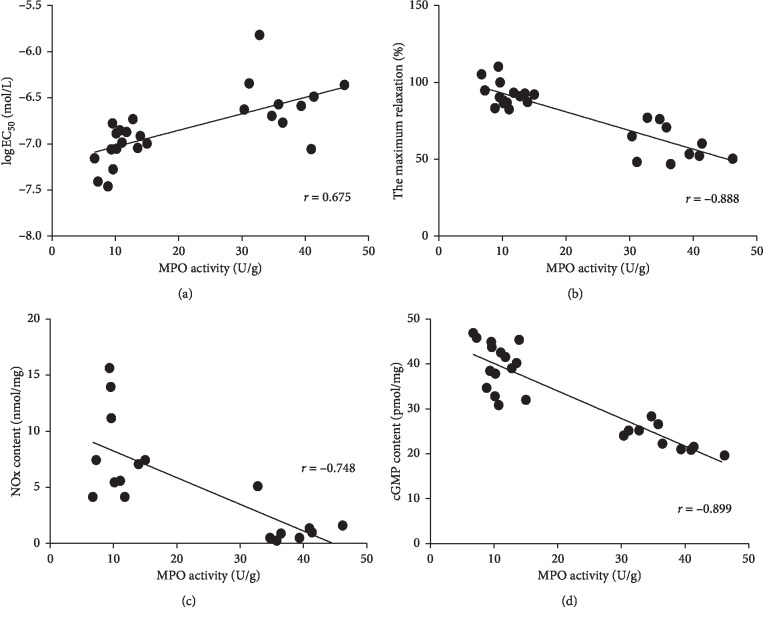
Correlation analysis of MPO activity with vascular endothelial function, NO_*x*_ content, and cGMP level in thoracic aorta rings between HC diet rats and after intervention with the PPAR*γ* agonist. There was a positive correlation between MPO activity and the log EC_50_ value of ACh-induced vasodilation (a); ACh-induced maximum vasodilatation (b), NO_*x*_ content (c), and cGMP level (d) showed negative correlation with MPO activity.

These studies aimed to further confirm the role of PIO in MPO, and its ability to improve vascular endothelial dysfunction in hypercholesterolemic rats. This study conducted an analysis of an association of vascular endothelial function, NO_*x*_ content, and cGMP level with vascular MPO activity in the hypercholesterolemia group and the PIO intervention group. The results showed that the log EC_50_ value of ACh-induced vasodilation positively correlated with MPO activity (*r* = 0.675, *P* < 0.01; Figure 4(a)); i.e., the EC_50_ value of ACh-induced vasodilation negatively correlated with MPO activity. We found that ACh induced a maximum state of vasodilation, which negatively correlated with MPO activity (*r* = −0.888, *P* < 0.01; Figure 4(b)). In addition, NO_*x*_ content and cGMP level negatively correlated with MPO activity (*r* = −0.748, *P* < 0.01; *r* = −0.899, *P* < 0.01; Figures 4(c) and 4(d)). It also suggested that, by inhibiting the vascular MPO activity, PIO could maintain the integrity of the vascular NO/cGMP/cGK signaling pathway, thereby protecting vascular endothelial function in hypercholesterolemic rats.

## 4. Discussion

Endothelial dysfunction caused by hypercholesterolemia is a prerequisite for the development and progression of atherogenesis and is associated with the occurrence of clinical events (i.e., unstable angina, acute myocardial infarction, sudden coronary death, etc.) in patients already presenting with atherosclerosis [12]. Although the mechanism of vascular endothelial dysfunction in hypercholesterolemia remains unknown, reversing or improving endothelial dysfunction is key to preventing atherosclerosis and subsequent ischemic heart disease.

Vascular endothelial cells regulate vascular tone by secreting an endothelium-derived relaxing factor (EDRF, namely, NO) with a vasodilatory effect, and an endothelium-dependent vasodilatory function that is often used to reflect endothelial function [13]. Some studies have reported that hypercholesterolemia can lead to vascular endothelial dysfunction [14]. In the present study, it was found that following administration of a recognized endothelium-dependent vasodilatory substance, i.e., ACh, the maximum vasodilatation of the thoracic aorta in hypercholesterolemic rats was significantly reduced. However, it was not significantly altered after administration of the endothelium-independent vasodilatory substance SNP. This observation suggests that endothelial dysfunction occurred in hypercholesterolemia, which was consistent with related reports.

NO is a key molecule that mediates endothelial function and causes vasodilation by activating the NO/cGMP/cGK signaling pathway [15]. MPO, which is derived from neutrophils, monocytes, and macrophages, reduces NO production and does so by modifying the NO donors L-Arg [16] and NOS [17]. Baldus et al. [18] reported that MPO circulating in the blood vessels decreases the bioavailability and biological activity of NO. Our study suggested that the vascular NO content in hypercholesterolemic rats was significantly reduced, and its biological activity (reflected by vascular cGMP level) was significantly decreased. Furthermore, this study found that vascular MPO activity in hypercholesterolemic rats was significantly increased, while vascular endothelial function was significantly reduced. Correlation analysis showed a significant negative correlation between vascular endothelial function, NO content, cGMP levels, and MPO activity. Moreover, after administration of the MPO inhibitor DDS in hypercholesterolemic rats, the vascular NO content and its biological activity were increased, with a concordant improvement in vascular endothelial function. These results suggested that MPO might be involved in vascular endothelial dysfunction in hypercholesterolemia and does so by affecting the NO/cGMP/cGK signaling pathway.

PPAR*γ* is a subtype of PPARs. Among the many synthetic ligands, TZDs are PPAR*γ*-selective agonists with high affinity, including troglitazone, rosiglitazone, and PIO, which are significantly effective in the treatment of type 2 diabetes and cardiovascular complications [19]. PPAR*γ* agonists can alleviate atherosclerosis by improving metabolic risk factors known to cause atherosclerosis and to reduce inflammatory factors in the arterial wall with an improvement in endothelial function seen in diabetic and hypercholesterolemic animals [20]. And, PIO attenuated palmitate-induced ER stress in macrophages, and the effect was reversed in the presence of PPAR*γ* antagonists [21]. Moreover, studies have confirmed that PPAR*γ* agonists can regulate MPO gene expression [10]. In the present work, we found that after PIO intervention in hypercholesterolemic rats, the endothelium-dependent vasodilatory substance ACh directed a significant improvement in the vasodilation of the thoracic aortic rings. By contrast, after administration of the endothelium-independent vasodilatory substance SNP, there was no significant difference in the vasodilatory effect between groups. This indicated that PIO could improve vascular endothelial function in hypercholesterolemic rats. At the same time, PIO could significantly reduce the vascular MPO activity in hypercholesterolemic rats and effectively reverse vascular NO content and cGMP level—observations that were similar to the effect seen with MPO inhibitors. In addition, the MPO activity of vascular tissues negatively correlated with vascular endothelial function, NO_*x*_ content, and cGMP level after intervention with the PPAR*γ* agonist. It suggested that PIO might improve vascular endothelial dysfunction in hypercholesterolemia by regulating vascular MPO activity via the NO/cGMP/cGK signaling pathway.

In addition, this study found that after administration of PIO, there was a significant decrease in blood lipid levels in hypercholesterolemic rats; thus, it was believed that the lipid-lowering effect of PIO might be associated with improved endothelial dysfunction. This protective pathway might be different from the abovementioned pathways that were shown to be mediated by regulation of MPO. Numerous clinical data have demonstrated that hypercholesterolemia is one of the most common complications of type 2 diabetes and is the leading cause of atherosclerosis, coronary heart disease, cerebrovascular accident, and ultimately the death of patients presenting with type 2 diabetes. Thus, drugs that can simultaneously treat coronary heart disease, diabetes, and hypercholesterolemia might have additional clinical utility and applications in the management of several conditions. Thus, it is of paramount clinical significance to explore the mechanisms of such drugs in future studies.

## 5. Conclusions

This study found that (1) MPO might provoke vascular endothelial dysfunction in hypercholesterolemic rats by reducing NO biological activity and impairing the NO/cGMP/cGK signaling pathway and (2) the PPAR*γ* agonist PIO might inhibit vascular MPO activity and increase NO bioavailability with the net result of reversing endothelial dysfunction in hypercholesterolemic rats.

## Figures and Tables

**Table 1:** Lipid profile in different groups (mmol/L).

Group	*n*	0 weeks			8 weeks		
		TC	TG	LDL-CHO	TC	TG	LDL-CHO
Normal	12	1.44 ± 0.21	0.86 ± 0.21	1.44 ± 0.18	1.39 ± 0.16	0.65 ± 0.14	1.26 ± 0.37
HC	16	1.27 ± 0.32	0.82 ± 0.33	1.31 ± 0.27	3.12 ± 0.29^*∗∗*^	1.61 ± 0.36^*∗∗*^	2.38 ± 0.21^*∗∗*^
HC + DDS	16	1.32 ± 0.27	0.99 ± 0.27	1.29 ± 0.32	3.59 ± 0.38^*∗∗*^	1.78 ± 0.39^*∗∗*^	2.53 ± 0.45^*∗∗*^
HC + PIO	16	1.30 ± 0.39	0.77 ± 0.31	1.37 ± 0.29	1.56 ± 0.20^##^	1.12 ± 0.29^##^	1.68 ± 0.33^##^

Values are expressed as mean ± SD. TC: total cholesterol; TG: triglyceride; LDL-CHO: low-density lipoprotein cholesterol; normal: normal diet group; HC: HC diet group; DDS: dapsone; PIO: pioglitazone. ^*∗∗*^*P* < 0.01 vs. normal diet group; ^##^*P* < 0.01 vs. HC diet group.

## Data Availability

The data used to support the findings of this study are available from the corresponding author upon request.

## Supplementary Materials

Supplementary Materials Supplementary Figure: the changes of MPO activity, log EC_50_ value of ACh-induced vasodilation, NO_*x*_ content, and cGMP level in the thoracic aorta tissue with the HC + DMSO group: (a) MPO activity; (b) log EC_50_ value of ACh-induced vasodilation; (c) NO_*x*_ content; (d) cGMP content. ^*∗∗*^*P* < 0.01 vs. normal diet group; ^#^*P* < 0.05, ^##^*P* < 0.01 vs. HC diet group; ^±^*P* < 0.05, ^±±^*P* < 0.01 vs. HC diet-group and + DDS group. *n* = 6–10 rats/group.
