# Supplementary material for: The Peroxisome Proliferator-Activated Receptor γ Agonist Pioglitazone Protects Vascular Endothelial Function in Hypercholesterolemic Rats by Inhibiting Myeloperoxidase
Source: Cardiol Res Pract. 2020 Jan 7;2020:1845969. doi: 10.1155/2020/1845969 (PMC7063881; doi:10.1155/2020/1845969)
Supplement: Supplementary Materials — Supplementary Figure: the changes of MPO activity, log EC50 value of ACh-induced vasodilation, NOx content, and cGMP level in the thoracic aorta tissue with the HC + DMSO group: (a) MPO activity; (b) log EC50 value of ACh-induced vasodilation; (c) NOx content; (d) cGMP content. ∗∗P < 0.01 vs. normal diet group; #P < 0.05, ##P < 0.01 vs. HC diet group; ±P < 0.05, ±±P < 0.01 vs. HC diet-group and + DDS group. n = 6–10 rats/group. [file 1845969.f1.pdf]

Supplementary Materials

Supplementary figure:

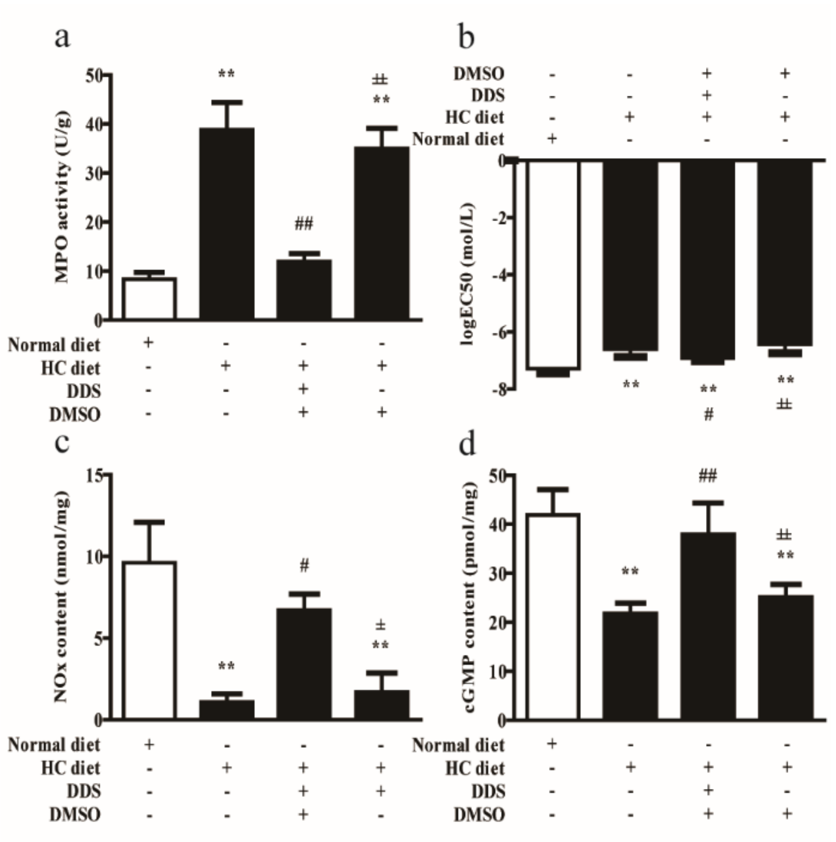

Supplementary figure Legend：

The changes of MPO activity, log EC_{50} value of ACh-induced vasodilation, NOx content and cGMP level in thoracic aorta tissue with HC+DMSO group. (a) MPO activity; (b) log EC_{50} value of ACh-induced vasodilation; (c) NOx content; (d) cGMP content. $^{**}P < 0.01$ *vs.* Normal diet group; $^{\#}P < 0.05$, $^{\#\#}P < 0.01$ *vs.* HC diet group; $^{\pm}P < 0.05$, $^{\pm\pm}P < 0.01$ *vs.* HC diet + DDS group. n = 6-10 rats/group.
